# Notes and News

**Published:** 1889-05-18

**Authors:** 


### Notes and News.

COLLECTED AND COMMUNICATED.

At Burton-on-Trent last week, a terrible death occurred from hydrophobia. A married woman, named Weston, was bitten by a cat that had been previously attacked by a mad dog. No notice was taken of the bite until a few days ago, when unfavourable symptoms developed, and medical aid was summoned, but the woman died in great agony.

Dr. Todd, of North Petherton, and the Rev. Prebendary Robinson, vicar of that place, attended a meeting of the North Petherton Farmers' Club, held at Bridgwater last Friday. The doctor and the vicar were driving home in a gig, when the horse bolted and dashed against some shop premises. Both of the occupants were thrown out, and on Dr. Todd being picked up it was found that his head was much injured. He died while being conveyed to the residence of a neighbouring surgeon.

We congratulate Mr. Shirley F. Murphy on his appointment by the County Council as medical officer for London. No one had greater experience or claims for this post, and we shall be disappointed if Dr. Murphy's election does not speedily bear fruit, in the direction of a marked improvement in the dwellings and sanitary arrangements of the poor throughout the metropolis. There is one thing the County Council should, however, do promptly, and that is to make the medical officer's salary more adequate to the work and responsibilities of the office by raising it to £1,500. The salary should never have been less than £1,500, and the ratepayers' best interests demand the speedy rectification of the initial mistake.

On Saturday, the 11th, Princess Louise presented the prizes to the Volunteer Medical Staff Corps, of which Sir William Guyer Hunter is the honorary commandant, in the Guildhall. The committee of the corps have made an appeal for financial aid in the erection of the new drill-hall, offices, etc., which it is forced to build in consequence of the extension of the Charing Cross Hospital, which will occupy the building now used as its headquarters. The object of the corps is to provide the Volunteer Force with something akin to the Army Medical Service—to train medical students to become efficient volunteer surgeons, and to extend the education and practice necessary to provide expert dressers and nurses in time of war. The corps has already rendered good services in time of peace, when the Lord Mayor's Show, the Jubilee Procession, or similar occasions have drawn large crowds together; and they also proved themselves useful in attending to the casualties incident on the riots among the unemployed.

The collection made by the United Orders Friendly Society Fraternity (Deptford and Greenwich District) on the occasion of the Palm Sunday church parade, has just been paid over to the "Dreadnought" Seamen's Hospital, Greenwich, and the Miller Memorial Hospital. The balance available for distribution after the payment of trifling expenses was £36 19s. 4d., and this sum has been equally divided between the two charities. The increase on the amount collected last year is due to the energies of Messrs. Everitt, Hill, West, and Pender.

The Manchester City Council have in hand a scheme for the removal of unhealthy dwellings and the providing of sanitary house accommodation for those compulsory dispossessed of their dwellings. That it was high time something were done was evident from the fact that the death-rate of children under five years of age in Manchester was 50 per cent. higher than the average throughout England. The scheme was carried by thirty-seven votes against four, and amidst much applause. Thoroughly carried out, the plan should greatly reduce the present death-rate and result in the saving of something like 3,000 lives per annum. A reference to the health map of Manchester distinctly proves the truth of M'Dougall's axiom that density and death go together.

The Jubilee wing for out-patients of Southampton Infirmary has been quietly opened without any public ceremony. It is a handsome and useful addition to the old building, by which it is connected by a covered passage, and contains an electrical room, provided with the newest instruments and also appliances for microscopic work. There is also an eye chamber, from which the light can be entirely excluded, which is very necessary during operations on this delicate organ. The committee has spared no expense in rendering this room complete in every respect, and the apparatus includes a special lamp and a case of new lenses, so there should be no necessity now for an eye hospital in the town. Mr. Mitchell, the architect, has presented a handsome clock to the institution.

Whenever it is proposed to build a fever hospital there is a great outcry from the public in the neighbourhood. They always fail to see that the hospital is built to prevent the spread of disease; they prefer to regard it as a disseminator of those horrid germs of which they have heard so much—and know so little! The question of an infectious hospital is to the fore in Huddersfield just now, so of course the local papers are deluged with letters full of useless suggestions. We would advise the people of Huddersfield and elsewhere to consider if fever is not more likely to spread if left in cottages and houses, where precautions cannot be taken and danger is not recognised; than if it is gathered into one spot where every care can be taken against infection, and where a competent staff will be ever on the alert against the insiduous germ?

At last the allegations against the Leicester Dispensary have been formulated, and were read at the annual meeting of governors on May 2. Briefly put, it is said the institution is conducted in a manner detrimental to its highest interests and to the interests of those for whose benefit it was created; that the manager has not discharged his duties in a straightforward or proper manner; that the quality of drugs and medicines dispensed from the institution has at times been far below the required standard of excellence; and that undue influence and pressure have been put upon medical men and others by those in whose hands the management of the dispensary rests. Certain irregularities of management are also alleged, and now the charges are plainly stated, the committee will have to take up the matter in earnest; it must either defend itself or die.

The following vacancies are announced this week, the amount of the salary in each case being given after the name of the institution:

**Resident Medical Officers.**

Royal Free Hospital. Senior Medical Officer.—£100, board, &c.
Bradford Infirmary. Junior House Surgeon.—£50, board, &c.
Adelaide Hospital, Australia. Medical Superintendent.—£500, board, &c.
London Temperance Hospital. Junior House Surgeon.—Board, &c.
Bristol Eye Hospital. Clinical Assistant.—£60.
Derby Infirmary. House Surgeon.—£100, board, &c.
North Staffordshire Infirmary. House Surgeon.—£100, board, &c.
Kensington Dispensary. Medical Officer.—£125, apartments, &c.

**Non-Resident Medical Officers.**

Taunton Union. Medical Officer.—£75.
Charing Cross Hospital. Dispenser.—£105.
National Dental Hospital. Anæsthetist and Lecturer.
University of Durham. Lecturer on Anatomy.—£300.

The young woman Guy, who it is alleged has been rendered insane through illegal detention for nearly a year in a shed at Broadway, Dorchester, has died in the Dorset County Lunatic Asylum. She has never recovered her reason since her escape from her supposed imprisonment. The proceedings against the man Burt, who is charged with having detained her, are not yet concluded.

The late squabble over a corpse at the Middlesex Hospital was a disgraceful proceeding. Two undertakers received orders to bury the body, and on both appearing at the hospital one was ordered away by the porter, whom he immediately assaulted. A summons was taken out, but in the meanwhile the remains of the unfortunate young woman had to be unburied for a day or two, until it was decided which of the two men should get the few paltry shillings paid for the interment.

Mr. Bros spoke very strongly at the Dalston Police Court lately on the treatment of persons found as wandering lunatics, who are now brought before police magistrates like common rogues or thieves. In this case a young married lady had been found wandering, and Mr. Bros would not allow her to be brought into court or placed in a cell. He saw her in the usher's room, and, on returning to the bench, said, "She is charged as though she had committed an offence, but she has committed no offence, and should be taken to the workhouse, where she would have medical and other proper attention. The police are only attending to orders; but (to Inspector Denning) I hope you will make my words known to the Commissioners."

On Saturday last Princess Christian paid a visit to Blackheath, and opened the new building of the Blackheath and Charlton Cottage Hospital. The neighbourhood was decorated with bunting, but the effect was greatly marred by the wet weather. At the principal entrance to the hospital Her Royal Highness was met by the reception committee, which included the Earl and Countess of St. Germans, the Marchioness of Dufferin and Ava, Sir Spencer and Lady Maryon Wilson, Dr. Clapton, the hon. physician; Mr. P. Cooper; Miss Westlake, the matron; and Mr. F. D. Lewin, the hon. sec. Her Royal Highness then proceeded to inspect the building, after which prayer was offered in the wards. Thence, entering the pavilion, the Royal visitor was escorted to a crimson-covered dais, decorated with choice flowers. Mr. H. Green, on behalf of the committee, presented Her Royal Highness with an address. Princess Christian then declared the hospital open. Purses were presented to Her Royal Highness by a number of little children, after which Her Royal Highness took her departure amid the cheers of the crowd who had gathered outside the hospital gates.

We understand that the Prince and Princess of Wales have shown the great interest they take in the Samaritan Hospital by promising to lay the memorial-stone of the new building in June or July. At the late dinner in aid of the building fund, over which Lord Charles Beresford presided, Dr. Granville Bantock said it was admitted by everyone that the great advance which has been made in the treatment of diseases peculiar to women within the last forty years has been due to the influence of special hospitals.

At a recent meeting of the Royal Asiatic Society of Japan a paper was read by Dr. Seymour on the hygiene of Japanese houses, in which he disproves the common idea that dwelling-houses in that country are very unhealthy. Amongst foreigners there is distinctly more illness in brick and stone houses than in the wood or frame houses, on account of the damp remaining in the walls of the brick houses, while it dries up almost immediately in the others. The remarkably small infant mortality amongst the Japanese shows that their houses are healthy and suited to their modes of life.

An esteemed correspondent reminds us that the report of the Nottingham General Hospital, quoted above, should be read in conjunction with some knowledge of the details of its management. The medical staff at Nottingham are generous with extras which they consider necessary to the welfare of their patients, and the nurses (lucky nurses!) have pudding every day, and fruit and green vegetables constantly. The ward floors and corridors are also washed daily, which is a costly process; but we are sure the good people of Nottingham who visit their clean bright hospital, nursed by healthy, happy women, and full of cheerful patients, do not desire a decreased expenditure. Where it is possible for the management to resist the paring-down process, and take the happy path between lavishness and meanness, there true economy is achieved. But that is a matter for the public; if they insist on the shaving system, committees must agree with them, for hospitals are public institutions.

The physicians of Suffolk General Hospital found themselves in an awkward position at the last meeting of governors. It was proposed to appoint three assistant surgeons to the hospital, and this question was, of course, of special interest to the present medical staff. The point was this: It had come to the notice of the committee that a great many of the out-patients did not see the out-patient staff, and if it was considered that at present there were sixty in-patients and 200 out-patients, it would be conceded that that was an enormous quantity of work to ask the medical officers to undertake. Dr. Macnab made an excellent speech, in which he pointed out that the matter was purely a doctor's question; no body of lay gentlemen could appreciate what the position of a hospital surgeon meant. If it was thought that the three medical officers had neglected their duties then they were bound to pass a vote of censure on them; but no gentleman had stated a distinct act of neglect. If he knew of one, now was the time to bring it forward. He could say for himself that he had never neglected his work at the hospital, and it had been his pride and pleasure to come there. He had kept a record of every visit and every operation which had been performed at the hospital since this year began, and was it not a hard thing, when the doctors were working there gratuitously, that this system of espionage should be exercised over them, working as they did for the love of the thing? The chairman submitted the resolution "that the medical staff be increased by the addition of three assistant surgeons," and it received the support of eighteen gentlemen, nineteen hands being held up against it.

